# Individual Differences in Thresholds and Consumer Preferences for Rotundone Added to Red Wine

**DOI:** 10.3390/nu12092522

**Published:** 2020-08-20

**Authors:** Jessica M. Gaby, Alyssa J. Bakke, Allison N. Baker, Helene Hopfer, John E. Hayes

**Affiliations:** 1 Sensory Evaluation Center, The Pennsylvania State University, University Park, Centre County, PA 16802, USA; jmg1069@psu.edu (J.M.G.); abc18@psu.edu (A.J.B.); anb22@psu.edu (A.N.B.); hxh83@psu.edu (H.H.); 2 Department of Food Science, The Pennsylvania State University, University Park, Centre County, PA 16802, USA; 3 Graduate Program in Neuroscience, The Pennsylvania State University, University Park, Centre County, PA 16802, USA

**Keywords:** specific anosmia, rotundone, wine tasting, individual differences

## Abstract

Rotundone is an aromatic compound found in the skin of some grapes (e.g., Shiraz, Noiret) that contributes peppery notes to wines made with these varieties. There may be a specific anosmia for rotundone, as some individuals are unable to detect it even at high concentrations, despite otherwise normal olfaction. This may affect perception of and preference for rotundone-containing wines. Here, we report rotundone detection thresholds (orthonasal *n* = 56; retronasal *n* = 53) and rejection thresholds (*n* = 86) in red wine for a convenience sample of non-expert consumers in Pennsylvania. Focus groups were conducted to better understand consumer attitudes and preferences for rotundone. Ortho- and retronasal detection thresholds were nearly identical (140 v. 146 ng/L). Roughly 40% of our sample was anosmic to rotundone, extending evidence for a specific anosmia to a North American cohort. As ortho- and retronasal thresholds were extremely similar, future work on rotundone can rely on orthonasal assessment. In our participants, added rotundone was generally disliked, and in focus groups, the concept of a ‘peppery’ wine was not appealing. Winemakers need to carefully consider biological and attitudinal segmentation when making and marketing peppery wines. Further work is needed to identify the genetic basis for this anosmia.

## 1. Introduction

Rotundone is a sesquiterpene with an aroma that is often described as peppery or spicy. It is responsible, in part, for the characteristic aroma of black pepper. Commercially, it is highly pertinent to the wine industry, as it is also found in some red wines such as Australian Shiraz or Pennsylvania Noiret. Rotundone was first isolated a little over a decade ago [1], and subsequent work has shown rotundone is found in the skins of certain grape varietals [2], including Shiraz/Syrah [1] and Noiret [3]. The concentration of rotundone in the skins of these grapes can be altered via viticulture practices [3,4,5]. Similarly, the amount of rotundone in wines made from these grapes can be controlled via enological practices, including how long the wines are left to ferment on the skins [2]. Noiret grapes are a hybrid varietal that grows well in the Eastern United States, and, anecdotally, these grapes are often used to make minimally peppery wines for the local market. Conversely, in Australian Shiraz, peppery aromas provided by rotundone can be highly desirable, as wines with a strong peppery character are able to command premium prices in the marketplace.

Prior work on rotundone perception also suggests it may exhibit a specific anosmia in some percentage of the population. Specific anosmia is the inability to smell a single odorant, despite an otherwise normal sense of smell. Based on extant data, roughly 1 in 4 or 1 in 5 individuals are unable to perceive rotundone [1,6]. In humans, the sense of smell depends on ~350 different olfactory receptors [7], each of which responds to a specific class of molecules (reviewed in [8]). Odor percepts arise from the pattern of activation across these different receptors (see [9] for a review). The specific receptors present in an individual’s nose and the number of each type of receptor are genetically determined, resulting in individual differences in odor perception [10,11]. That is, some individuals may lack a functional variant of a certain receptor and be unable to smell molecules that would bind to that receptor, while still having an otherwise normal sense of smell. Multiple compounds have been identified as showing specific anosmias in the population (e.g., [12,13]). For a few of these, like the smoky odor of guaiacol or the floral note of beta-ionone, a specific allele responsible for the anosmia has been identified [14,15]. Biologically driven differences in the perception of some compounds can have drastic impacts on food and beverage choice [16,17], and are therefore both nutritionally and commercially relevant.

Because rotundone was isolated relatively recently (circa 2008) and is typically present in only a few wine varieties, studies examining the perception of this compound are fairly limited. In the first study from Australia, one-quarter to one-fifth of participants were anosmic to the compound in red wine (9 of 47) and water (12 of 49). Among the responders, the orthonasal detection threshold was estimated to be 8 ng/L (in water) or 16 ng/L (in red wine), while anosmic individuals were unable to detect rotundone even at concentrations up to 4000 ng/L [1]. This specific anosmia was subsequently confirmed in a French study, which estimated ~31% of participants could not detect rotundone at 200 ng/L (i.e., a concentration well above threshold for responders) using both orthonasal and retronasal (in mouth) assessment [6].

Given the popularity of Australian Shiraz wines that contain rotundone, we wondered whether there might be a market in Pennsylvania for wines containing moderate to high rotundone concentrations. Previous research suggests that rotundone in wines can be polarizing. Geffroy and colleagues [6] reported that consumers in France could be separated into three segments: those who consistently prefer the control wine over rotundone-spiked wine, those who prefer a moderate concentration of rotundone (below 46 ng/L), and those who enjoy high concentrations of rotundone (>94 ng/L). Further, participants’ perception of added rotundone was in line with their previously declared preference for peppery notes in wine, with more experienced and older consumers belonging largely to the latter two groups. However, in this French study, most participants were familiar with peppery notes in wine. Peppery notes are not particularly common in Pennsylvania wines, and the most well-known wines from this region of the country are white wines, including Riesling and Gruner Veltliner. Therefore, we were interested in how Pennsylvania consumers, who might be unfamiliar with peppery wines, would respond to added rotundone. Accordingly, we employed mixed methods (i.e., quantitative sensory tests and qualitative focus groups) to more fully understand the consumer experience.

We had four aims that were tested in three separate experiments. First, as perception of rotundone has not been previously studied in a North American sample, and because prior reports used different methods to estimate thresholds, we determined the detection threshold of rotundone in red wine in a convenience sample of wine consumers. Secondly, we estimated the percentage of our participants who would be anosmic for this compound. As we began working with rotundone in our laboratory, we also noted rotundone appeared to be less intense when sniffed orthonasally than when assessed retronasally by swishing rotundone-spiked wine in the mouth. Given this informal observation, and the discrepancy in delivery method between prior studies [1,6], our third aim was to directly compare ortho- and retronasal detection thresholds for rotundone in red wine using participants who had been drawn from the same population. Finally, we wanted to assess consumer reactions to rotundone-containing wines, both quantitatively via a rejection threshold for rotundone added to red wine, and qualitatively, in consumer focus groups.

## 2. Materials and Methods

### 2.1. General Overview

Participants were recruited from an existing database of 1200+ individuals maintained by the Sensory Evaluation Center on the main campus of The Pennsylvania State University (Penn State), a large land grant university located in rural central Pennsylvania. Study qualifications included the following: not pregnant or breastfeeding, nonsmoker, no food allergies, no history of choking or difficulty swallowing, no known smell or taste defect, no self-reported history of alcohol dependency or religious aversion to consuming alcohol. All procedures were approved by the Penn State University Institutional Review Board (IRB; protocol #00011912). All participants who visited the laboratory received a small cash incentive for their time. All quantitative data were collected using Compusense Cloud, Academic Consortium (Guelph, ONT) in individual sensory testing booths under white light (5000 k LED) located overhead. Qualitative data were collected by an experienced moderator (AJB) in a focus group facility around a conference table with comfortable chairs and overhead lighting. All spaces were climate controlled.

### 2.2. Study 1–Detection Thresholds

A total of 109 participants were recruited for a single test session. Upon arrival to the laboratory, they were randomized into one of two conditions in a pairwise fashion: roughly half of participants smelled (sniffed) the wine samples but did not taste them, and the other half were asked to taste the samples by mouth before spitting them out. Hereafter, for convenience and readability, these two conditions will be referred to as the *orthonasal* condition and the *retronasal* condition (with the caveat that the second condition is not purely retronasal in nature, as taste and chemesthetic inputs are also present when sampling via the mouth).

We presented rotundone in red wine rather than water for increased ecological relevance; that is, this compound is typically encountered in wine, and prior work shows that threshold estimates can differ substantially between water and wine [18]. Within a single visit, each participant was given 5 separate triads of samples, for a total of 15 samples; no replicates were obtained. Participants provided informed consent twice, once for the screener to determine eligibility and again for the study itself.

### 2.3. Study 1–Participants

The orthonasal condition was completed by 56 participants (12 men, 44 women) while the retronasal condition was completed by 53 participants (10 men, 40 women, and 3 not reported). There was no overlap across conditions: participants only completed orthonasal assessment or retronasal assessment, but not both. The modal respondent was female who self-reported as Caucasian and was 25–30 years old (Table 1). Additional details are provided in Supplementary Table S1.

### 2.4. Study 1–Stimuli

Based on prior reports (i.e., an estimated detection threshold of 16 ng/L in red wine, and 31% of the sample being unable to detect rotundone at a fixed concentration of 200 ng/L in water), we selected a concentration range above and below these values. To minimize fatigue, we used 5 concentrations of rotundone: 0.2, 2, 20, 200, and 2000 ng/L. Stock solutions of rotundone in ethanol (95% USP grade ethanol, Koptek, King of Prussia PA) were prepared and then added to 4 L jugs of red wine. Based on standard practices for determining the influence of specific compounds on wine perception (e.g., [19]), we chose a mass-produced, fault-free wine (Carlo Rossi Burgundy, Carlo Rossi Vineyards, Modesto, CA, which is owned by E.J. Gallo) as the base for our stimuli, and then added rotundone at appropriate concentrations. This base wine is available in 4 L jugs, which allowed us to create a single batch of each rotundone concentration for maximum consistency; these jugs were used for all participants in both the orthonasal and retronasal groups. This dry wine was selected by 2 experienced wine researchers who tasted it to ensure it was fault free with a clean background; a neutral base red wine was used here because we desired a base wine without any distracting aromas from oak or high ethanol, as they might otherwise distract from or overpower the rotundone. Carlo Rossi Burgundy was specifically chosen for its overall neutrality; this specific wine has been used previously as a base wine for creating references for a trained panel (e.g., [19]). It is also widely available from a large producer, thereby facilitating future reproducibility of this work. The ethanol content was 12% (v/v); other parameters were not available, but they were within the legal limits for commercial red table wine in the US (27 CFR § 4.21): volatile acidity less than 0.14 g/100 mL, total sulfur dioxides over 10 ppm and less than 350 ppm (27CFR 4.22(b)(1). Readers should note that the descriptor *‘Burgundy’* is merely a historic trade name Carlo Rossi has used for many decades, and it should not be used to infer any stylistic similarities to Pinot Noirs from France. Rotundone was kindly provided by Dr. Markus Herderich and the Australian Wine Research Institute (Glen Osmond, South Australia). Concentrations were as prepared by the research team; no attempt was made to quantify these via instrumental chemical analysis. In bench testing by the research team, the wines with added rotundone (at higher concentrations than detection threshold) were perceptibly peppery whereas the unspiked wine was not.

### 2.5. Study 1–Psychophysical Task Completed by Participants

Detection threshold estimates were determined in accordance with ASTM Method E679-04 (“Standard Practice for Determination of Odor and Taste Thresholds by a Forced-Choice Ascending Concentration Series Method of Limits”). Briefly, participants completed a series of triangle tests, where every triad contained one spiked sample and two blank samples. Triads were presented in a fixed order so that the spiked sample increased in concentration across triads to minimize adaptation and fatigue. The order of samples within a triad was randomized. A minimum break of 90-s was enforced between triads. Each sample consisted of 20 mL of wine (spike or control) in a standard clear, colorless ISO wine-tasting glass (ISO 3591). For each triad, participants were asked either to sniff (orthonasal) or taste (retronasal) the three samples in the order presented on the tray, and to identify which sample was the most different among the three (i.e., standard triangle test instructions). In the retronasal condition, participants who sampled the wine by mouth were instructed to expectorate the wine after tasting. After each triad, participants were asked to use water to cleanse their palates before continuing. Participants received the first three triads on a single tray, and then exchanged the tray to receive their fourth and fifth triads. Responses were given via computer using Compusense Cloud. In total, testing took approximately 20 min.

### 2.6. Study 1–Threshold Definition and Data Analysis

For the triangle test at each concentration, we tallied whether or not the participant got the triangle test correct. We used these data to calculate a best estimate threshold (BET) for each individual. To do so, we used the standard ASTM E679 decision rule, with two modifications. Per the standard method, for most individuals, we defined their BET as the geometric mean of the first concentration where the participant got all subsequent levels correct, and the next concentration (level) down. For the two participants who were correct at the lowest concentration presented, their threshold was calculated as the geometric mean of the lowest concentration given (0.2 ng/L) and the next hypothetical concentration down (i.e., 0.02 ng/L), in accordance with the standard ASTM method. However, for 4 participants (of 109), an alternative decision rule was used to define individual BETs. Specifically, these four individuals all got three lower concentrations in a row correct before getting a higher concentration incorrect; because the probability of getting three in a row correct by chance is quite low (3.7%), we reasoned these individuals may be sensitive to rotundone and that the incorrect answers at higher (and nominally easier) concentrations were probably due to adaptation. For these 4 individuals, their BET was calculated instead as the geometric mean of the first concentration where the participant began the run of three correct, and the next concentration (level) down. Finally for the non-responders (i.e., those who got the triangle test at the highest concentration presented wrong, and did not have a run of three lower concentrations correct), the BET was imputed as the geometric mean of the top concentration (2000 ng/L) tested and the next theoretical concentration that would have been tested (20,000 ng/L); this value was only used for visualization in histograms and was not included in calculated means or any statistical testing.

Using the individual BETs for the responders, we calculated a group threshold estimate as a geometric mean. This was done by calculating the arithmetic mean of the logged BETs, and then taking antilog of this value, again consistent with the standard E679 approach. To formally test for differences in thresholds across the two conditions (orthonasal versus retronasal) in the responders, we used an unpaired t-test on logs of the individual BETs.

As an alternative analysis, we also used regression of the individual responses at each concentration to visualize and calculate group thresholds, using the graphical method of Lawless [20] as modified by Perry and colleagues [21]. Briefly, individual responses at each concentration were coded as 0 for incorrect and 1 for correct, and a regression line was fit to these points. The resulting line was used to determine the logged concentration where 67% performance was achieved by the group (i.e., halfway between perfect performance of one and chance performance of one third). This value was then antilogged to estimate the group threshold in ng/L for that condition. As discussed by Perry et al. [21], this approach provides a threshold estimate that (i) is adjusted for chance, (ii) does not vary with number of participants, and (iii) does not require specialized analysis software.

### 2.7. Study 2–Rejection Thresholds

We conducted a rejection threshold task using standard methodology [21,22]. Briefly, participants (*n* = 86) were presented with six pairs of samples: in each pair, one glass contained an unspiked wine sample and the other contained wine spiked with rotundone. Participants sampled each pair by mouth, spit out the wine, and indicated which they preferred in a forced choice task (additional detail below). Each participant assessed 12 samples total; no replicates were obtained.

### 2.8. Study 2–Participants

Participants were recruited as described for study 1. Due to logistical constraints, study 2 was conducted approximately one month prior to the detection threshold study, so we did not pre-screen for rotundone responder status. A total of 86 participants (49 women) completed the study; of the 86, 35 also completed the detection threshold study described previously. Again, the modal respondent was a Caucasian woman, 25–30 years old (Table 2). See Supplementary Table S2 for additional details.

### 2.9. Study 2–Stimuli for Rejection Thresholds

We selected a concentration range that includes both a) realistic concentrations of rotundone naturally found in Pennsylvania Noiret wines (approximately 100–200 ng/L based on [3]) and b) values above and below the previously reported detection threshold of 16 ng/L in red wine [1]. Accordingly, we used 6 concentrations: 3.75, 7.5, 37.5, 75, 375, and 750 ng/L of rotundone. Stock solutions of rotundone in ethanol (95% USP grade ethanol, Koptek, King of Prussia PA) were prepared and then added to 4 L jugs of a neutral, fault free red wine (Carlo Rossi Burgundy, Carlo Rossi Vineyards, Modesto, CA, USA) to create single jugs of each rotundone concentration, which were used for all participants. Wines (20 mL) were served at room temperature in clear, colorless ISO wine-tasting glasses (ISO 3591), each covered with a clear plastic cap and labeled with a three-digit blinding code. Samples were presented with three pairs to a tray, and participants were instructed to exchange trays halfway through the test to receive the rest of their samples.

### 2.10. Study 2–Rejection Threshold Task Completed by the Participants

Rejection thresholds were determined via the method described by Perry et al. [21]. Here, participants were asked to taste six pairs of wines (one spiked and one control) and indicate which sample they preferred in each pair. The pairs were presented in a fixed order so that the spiked sample increased in concentration across pairs to minimize adaptation and fatigue; the order of samples within a pair was randomized. Participants were provided with drinking water and instructed to place the entire sample in their mouth and swish, then expectorate, and rinse their mouth between samples; a 90-s break was enforced between pairs. Responses were collected via computer running Compusense Cloud. In total, testing took about 20 min.

### 2.11. Study 2-Rejection Threshold Definition and Data Analysis

To first determine responder/non-responder status, we adapted the ASTM E-679 decision rule for detection thresholds. Specifically, we examined responses for the three highest concentration levels (75 ng/L, 375 ng/L, and 750 ng/L). As prior data suggest detection thresholds for this compound are somewhere between 16 ng/L and 145 ng/L, we expected participants who would able to detect the compound would be able to do so within this range of concentrations. Conservatively, we defined participants who displayed consistent preferences for all three of the top 3 concentrations to be responders, regardless of the direction of the preference (i.e., blank or spike); the chance probability of selecting the same sample (either blank or spike) in three successive 2AFC tests purely at random is relatively low (12.5%). For this analysis, individuals who did not show this degree of consistency were considered to be non-responders. Using this strict decision rule, only 34 of 86 participants were considered consistent responders. We then estimated a rejection threshold within the consistent responders using the method of Perry et al. [21], where preference for the spike was coded as 1. A priori, crossing a criterion of 0.75 would indicate the threshold concentration where the group preferred the rotundone-containing wine, while crossing a criterion of 0.25 would indicate the concentration where the group preferred the unspiked blank.

### 2.12. Study 3–Focus Groups

To confirm the polarizing nature of rotundone highlighted in Study 2, we next conducted focus groups with local wine consumers. Three focus groups with a total of 25 participants (6 men, 19 women, ages 21–70, median age 32) were conducted to gain further qualitative understanding of consumer preferences and perceptions for rotundone-containing wines. Each group lasted 90 min and was comprised of 7–10 participants and led by an experienced moderator who followed a structured guide. The primary aim of these focus groups was to better understand the distaste consumers expressed for rotundone given on our rejection thresholds (see Study 2 Results below). Peppery wines are not commonly produced in Pennsylvania, and we did not expect casual wine consumers in Pennsylvania to be especially familiar with the concept or flavor of rotundone. Therefore, our focus group discussions were geared towards two objectives. The first was understanding whether the concept of a peppery wine was appealing to our consumers and, if so, in what context. The second objective was to explore how people might perceive wines with peppery notes given that there are individual differences in rotundone sensitivity.

### 2.13. Study 3–Participant Recruitment

Participants were recruited from the same database described previously. For the focus groups, study qualifications consisted of the following: 22–70 years old, no food allergies or sensitivities to alcohol, do not avoid alcohol for medical or ethical reasons, not pregnant or breastfeeding, regularly consume red wine, and were articulate in their ability to discuss wine (assessed via open ended online question about wine preferences). We did not assess participants for rotundone anosmia.

### 2.14. Study 3–Focus Group Discussion and Guided Sample Evaluation

Prior to the start of focus groups, the moderator read aloud a description of activities the focus groups would entail and described risks and benefits of the research. Participants gave verbal consent before proceeding with the research. All procedures were approved by the Penn State University IRB (protocol #13930).

To initiate the conversation, consumers discussed generally their motivations and personal processes for choosing wines to purchase. They were then prompted by the moderator to describe their favorite wines and attributes that they considered attractive in wines. Any mentions of taste, flavor, or other sensory characteristics (especially terms that would relate to the concept of ‘peppery’) were probed further to understand how consumers described desirable wines. Consumers were then led through a series of wine tastings, followed by discussion.

Over the course of each focus group session, participants tasted five different wines presented as 20 mL samples in ISO tasting glasses with clear plastic caps. Wines were presented with three-digit blinding codes. Wines were presented in a specified order (described below) rather than a randomized order. We acknowledge this choice could result in some order bias, but this was done intentionally to minimize sensory adaptation and to facilitate the discussion objectives with ease. Participants first tasted a Noiret wine naturally high in rotundone (210.6 ng/L). This wine was made by Penn State personnel under the supervision of a trained experienced enologist. Participants were asked to taste (and expectorate) the wine, then rate how much they liked the wine and to write down reasons for that rating. A simple rating scale of 1 to 5 with 1 being highly disliked and 5 being highly liked was used; this scale was not meant to collect quantitative data, rather it was included to facilitate discussions of all wines by providing a common framework for all participants to use for all wines. After all participants finished rating the wine and recording their responses, each participant shared their rating and discussed the rationale behind it. A major purpose of this sample was to identify the words lay consumers would use to describe a red wine with relatively high rotundone content.

After fully discussing that wine, participants were presented with two samples of neutral defect free red wine (the same Carlo Rossi Burgundy used in Studies 1 and 2). The first sample was unaltered while the second had been spiked with 200 ng/L rotundone. Participants were asked to taste the unspiked blank first and write down their ratings with justification, as before. After fully completing their assessment, participants were asked to repeat the procedure for the spiked wine sample. By providing participants with a control and spiked sample, we could isolate rotundone as the difference between the two wines. Once all participants had assessed both wines, ratings and justifications were discussed. Per the planned discussion guide, the term ‘peppery’ was to be immediately probed when it came up, but if pepperiness (the most common description for rotundone flavor in the literature) had not yet been mentioned, the moderator brought it up during this discussion segment. This was done by noting to participants that the spiked wine was often described as peppery and then asking participants if they had noticed that flavor or not. Then consumers were asked if they had ever had wines that they would describe as peppery. Probes included whether participants sought out peppery wines and for which occasions, how participants would respond to a wine described as peppery, and finally, how participants would personify a peppery wine.

Because many sweeter wines are commercially successful in Pennsylvania, we also wanted to include an example of a sweet red wine with added rotundone. We presented another pair of Carlo Rossi Burgundy wines, again with one sample spiked with 200 ng/L of rotundone. For this pair, both wines were back-sweetened with 3.5% *w*/*v* sucrose (purchased at retail from a local supermarket). Participants followed the same procedures as before for ratings and discussions. After trying all the wine samples, participants were asked to rank the four Carlo Rossi samples from most to least preferred, to share their rankings, and to discuss any items that they felt had not yet been addressed.

## 3. Results

### 3.1. Study 1–Detection Thresholds

**Figure 1:**
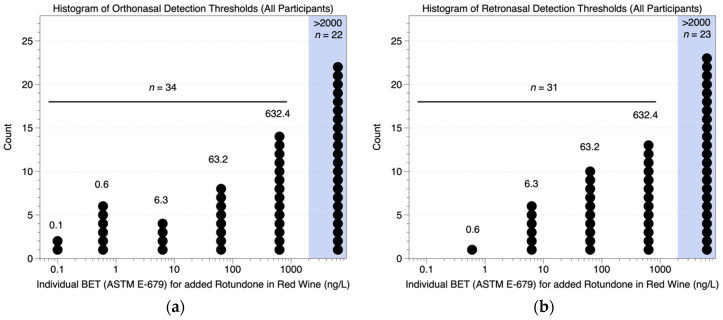
Estimated detection thresholds for rotundone in red wine assessed via the nostrils (orthonasal; (**a**)) or via mouth (retronasal; (**b**)) using the standard ASTM E679 method.

Across all participants, we observed marked variation in individual thresholds (BETs), as shown in Figure 1. The proportions of responders and anosmic individuals, i.e., those that didn’t detect rotundone at the highest concentration presented (2000 ng/L), were similar for both routes of odorant delivery: of 56 participants who smelled the samples (Figure 1a), 22 were anosmic (i.e., 61% were responders), while of 54 participants who sampled the wines by mouth (Figure 1b), 23 were anosmic (i.e., 57% were responders). After excluding anosmic individuals, the estimated threshold for the responders (i.e., the geometric mean of the individual BETs) was 37 ng/L (±11 SD) for the orthonasal condition and 73 ng/L (±21 SD) for the retronasal condition. Critically, these values were not significantly different from each other (t_63_ = 1.01; *p* = 0.30, on logged BETs), suggesting that route of delivery does not influence the detection of rotundone.

**Figure 2:**
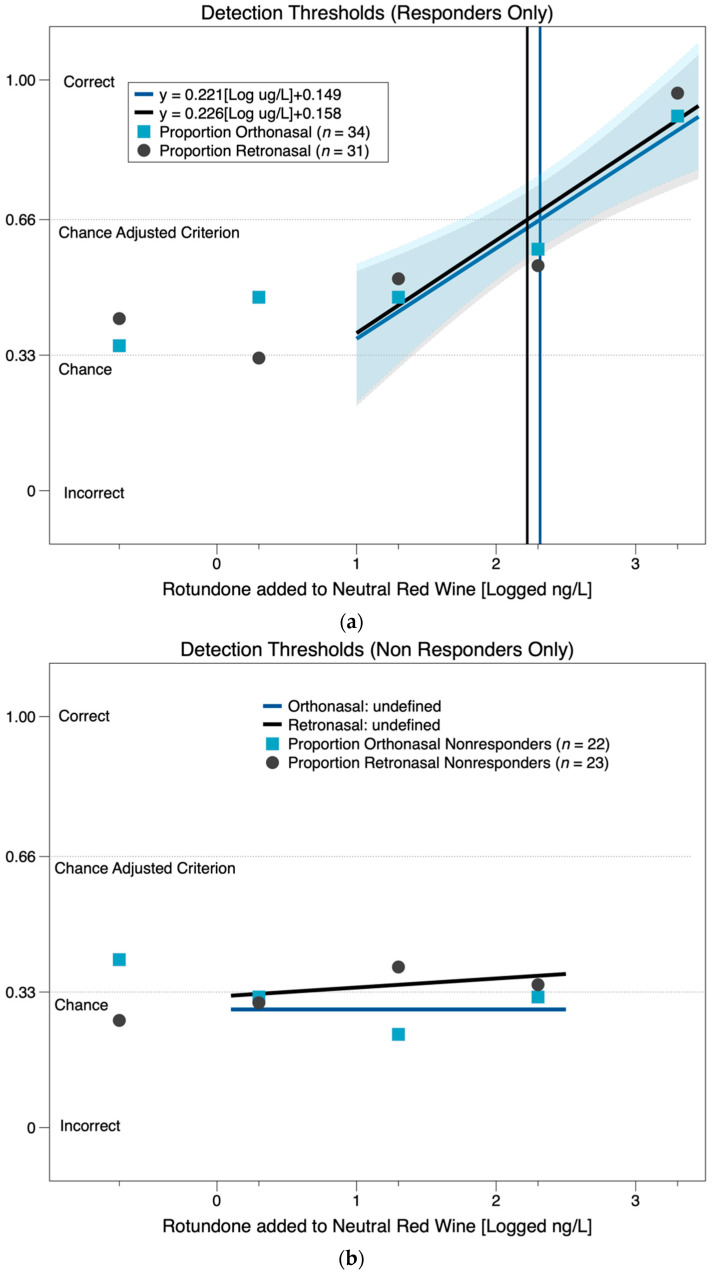
Estimated detection thresholds for rotundone in red wine using the graphical method, for responders (**a**) and non-responders (**b**) using the graphical method of [20], as modified by [21].

From the graphical approach (Figure 2), we also see clear evidence of individuals who are able to detect rotundone (Figure 2a), and of individuals who are anosmic (Figure 2b). After excluding those who are unable to detect the rotundone, it again appears that the route of delivery does not influence the detection of rotundone, as the estimated thresholds for both conditions are very similar (140 versus 146 ng/L). From the confidence interval of the graphical method, the lower and upper bounds of the orthonasal estimate were 39 and 832 ng/L. For the retronasal group, the lower and upper bounds of the estimate were 58 and 473 ng/L. As the confidence intervals overlap substantially, this approach confirms that threshold does not differ by mode of delivery.

### 3.2. Study 2–Rejection Thresholds

**Figure 3:**
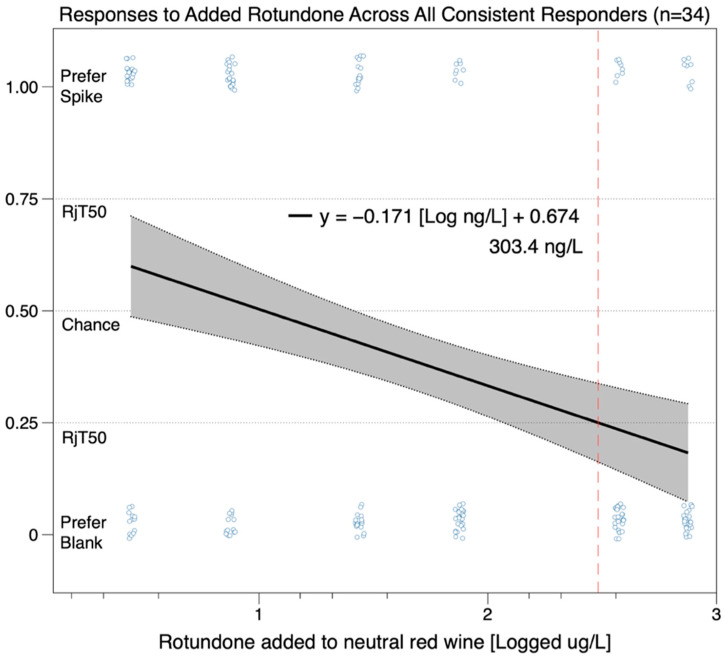
Estimated rejection threshold for all 34 participants who had consistent responses to the top three rotundone concentrations (75 ng/L, 375 ng/L, and 750 ng/L) added to red wine.

Using the criterion of responder/non-responder status for rejection threshold calculation as described above, of the 34 consistent responders (12 men and 22 women), only 8 individuals preferred the wine spiked with rotundone. This suggests that the peppery aroma from rotundone was not desirable to the group as a whole. When a regression line was fit to the preference data from all consistent responders (Figure 3), the line had a negative slope, indicating that the group of consistent responders did not like wine with added rotundone. From Figure 3, we can estimate the rejection threshold to be ~300 ng/L; however, this value should be interpreted extremely cautiously given the extremely clear segmentation across the group of responders (i.e., 8 preferers and 26 non-preferers).

**Figure 4:**
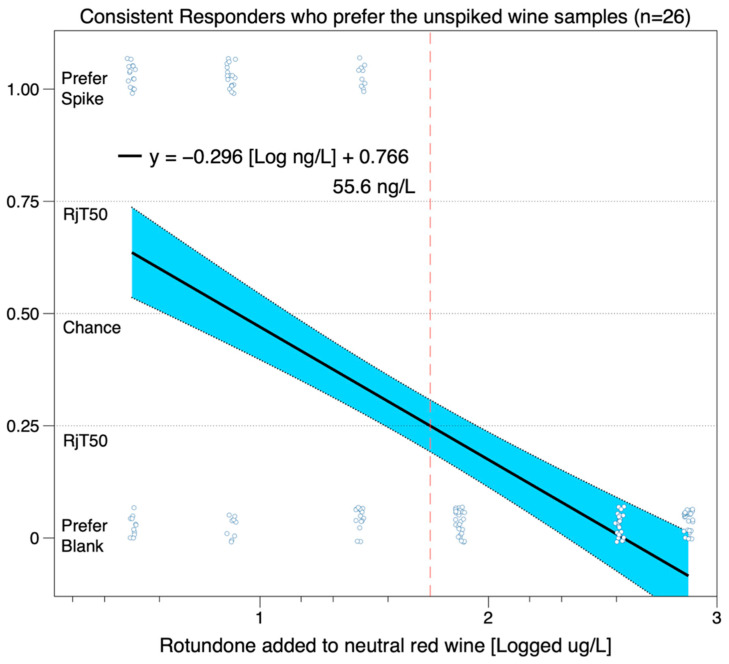
Estimated rejection threshold for consistent participants, after removing the 8 consistent participants who preferred the rotundone-containing wines.

However, if the 8 individuals who consistently liked rotundone at the top three concentrations are removed from the dataset, and a rejection threshold is recalculated in the 26 consistent responders who prefer the unspiked samples at the highest three concentrations, then the rejection threshold for rotundone is closer to ~56 ng/L, as shown in Figure 4.

Given the very low *n*, we did not attempt to calculate an exact threshold for the 8 consistent responders who liked the rotundone spiked samples, but there was a clear breakpoint between 37.5 ng/L and 75 ng/L, as values below 37.5 ng/L were essentially random (3 to 5 choices of 8), whereas at 75 ng/L and above, there was a striking preference (8 of 8 for all three concentrations). Collectively, these suggest that individuals who consistently select the same sample at the top three concentrations (and presumably can perceive rotundone) find it very polarizing. Compellingly, this polarization emerges as soon as they can perceive it, as our rejection thresholds in Study 2 are in a similar range to the detection thresholds estimated in Study 1.

### 3.3. Study 3–Focus Group Discussion

Results from focus groups provided information about how consumers perceive peppery wine, possible reasons consumers may like or dislike peppery flavors in wine, and information and meaning peppery flavors may communicate to consumers.

When tasting rotundone-containing wines (both the Noiret high in endogenous rotundone, and the Carlo Rossi base wine spiked with rotundone), many participants identified an additional flavor, but many had trouble naming it. For example, two participants said:

*‘There’s something in there different than just the fruit.’*

*‘There’s something there, but I can’t tell what it is.’*

Very few participants used the term peppery to describe the wines prior to the introduction of the concept by the moderator. Instead, participants used a mix of descriptors including *spice* or *spicy*, *earthy, woody*, and *strong* or *biting*. In confirmation of the rejection threshold data in Study 2, most but not all participants responded more positively to wines *without* rotundone. Most participants did not like the Noiret wine we presented, and when asked to directly compare spiked and unspiked samples of the Carlo Rossi Burgundy (both dry and backsweetened), participants generally preferred the unspiked wines. Participants cited disliking the aromas, flavors, or aftertastes of rotundone-spiked wines, often describing these wines as ‘strong’, ‘biting’, or ‘bitter’. Participants mentioned relatively higher fruit or fruity flavors and perceived sweetness as reasons for preferring the unspiked wines.

Example comments from participants when describing rotundone-spiked wines included:

‘It was very, very strong, just smelling it. My nose actually started to tingle a bit, which was weird. Near the end, I got that woody taste, which I also do not like.’

‘I thought that it didn’t really have any fruity taste at all. It just seemed really dry to me, and bitter.’

“[It] had a little bit more dimension but it was also spicier, which I don’t like.”

On the other hand, there was a subset of participants who responded positively to wines with added rotundone. These participants described the rotundone-spiked wines as more balanced and noted an appreciation for the spice notes. These participants tended to prefer drier wines and tended to comment that the flavors balanced any sweetness in the wine. Examples comments from these participants when describing rotundone spiked wines included:

‘The aroma is… I don’t know how to describe it. It’s more mature, and it’s not just fruit.’

‘It reminded me of Christmas. I felt like I felt, well, I tasted spices—cinnamon, nutmeg.’

‘Because [it] was slightly drier, I probably would go for that one.’

In addition to understanding consumer perception of wines containing rotundone, we also sought to understand consumer perceptions of the idea or concept of peppery wines. In general, the concept was not particularly compelling to our participants, with most saying that they would not seek out peppery wines for themselves. Those who did like the idea of peppery wines felt that they were most suitable to be paired with food or consumed slowly or in smaller quantities. Many mentioned beef as a possible pairing.

*‘Well, I think in the evening if you’re just having a glass of wine with the TV or a fireplace going, that’s different than having it with your meal. I think the heavier, drier, more peppery wines for me, better with food.’*

*‘You’re not taking a peppery wine to a college party or something. This is something you’re sipping with your family, or alone in your study, type thing. It’s a sipping wine, it’s a drinking at your home by the fireplace, type wine. Not a, “Woo! Let’s have another glass of peppery wine.”’*

Participants also indicated that it may be a more specific type of person who liked peppery wines, so it wouldn’t necessarily be appropriate for large parties or gatherings.

*‘I wouldn’t buy it for a party because it seems like a pretty polarizing wine, if that makes sense.’*

Asking consumers to describe a peppery wine as a person (i.e., to personify the wine) provided insights into what the term peppery might connote in a wine description or marketing campaign. The term “peppery” seemed to communicate one of two concepts, with the first being a very bold and flavorful wine, with these comments illustrating what participants envisioned:

‘I would think maybe a little bit more of a dominating personality. Well, you call someone spicy. You know what I mean like ‘Well, Spicy!’ So I don’t know. I feel like it’s definitely more, it’s a bolder choice. I think that person is probably a little just bolder in general.’

‘Well, first of all, I thought of it as a person that I used to work with. It is a woman and she doesn’t have a lot of boundaries. And I don’t mean that in a bad way. She’s a fun person to be with. She’s always cracking jokes and she’s just really lively.’

‘I agree with their descriptions, but I would add to that, just a little bit abrasive… someone who is very bold and says what they think.’

When asked to personify the wine, an equal number of participants instead envisioned a traditional, masculine, sophisticated wine. For example, participants said:

‘I’m picturing an older gentleman sitting in his study with a cigar.’

‘A very educated intellectual that’s very reserved and sophisticated.’

## 4. Discussion

The four aims of this project were to determine detection thresholds for rotundone in red wine via both ortho- and retronasal assessment, to assess the percentage of participants in our sample who display a specific anosmia for rotundone, and to determine preferences for and perceptions of peppery aromas and flavors in wine among those who could perceive rotundone.

The orthonasal detection threshold and the retronasal detection threshold were not significantly different from each other (37 and 73 ng/L, in the ASTM method and 140 and 146 ng/L with the graphical method). Approximately 40% of our participants were anosmic to rotundone at the highest concentration (not counting those who exhibited evidence of adaptation).

Regarding the slight differences between the two separate analysis methods (i.e., ~50 versus ~140 ng/L) for determining detection thresholds, Lawless previously suggested the ASTM method may yield lower threshold values than the graphical method, particularly if some participants in the sample exhibited adaptation at higher concentrations. Our results support this prediction, both overall, and because a handful of our participants appeared to show specific evidence of adaptation. In general, we prefer the graphical method over the ASTM method for several reasons. First of all, it is less sensitive to issues of adaptation and one-off mistakes (i.e., incorrect answers) from panelists who are momentarily distracted (see [20]). Second, this method does not require hand-coding to determine individual BETs, which is both laborious and potentially subject to coding errors. Particularly for experiments with very large numbers of participants and/or those employing many concentration levels, the graphical method has a very clear advantage in the efficiency of data processing and analysis. Here, our threshold estimate using the graphical method is somewhat higher than the previously reported group mean orthonasal threshold of 16 ng/L [1]. Critically, however, that study was performed among individuals employed at a wine research institute located in a region known for peppery wines, so the difference may reflect non-random sampling bias of sensitive individuals, or a simple familiarity effect (discussed below). On the other hand, when using the graphical method, the lower end of our confidence interval (39 ng/L) is not especially different from the values reported by Wood and colleagues [1].

Here, we found a slightly lower percentage of responders in our sample (i.e., more anosmics) than either of the two previously published studies [1,6]. This could be due to several factors. It is possible that there are simply regional variations in the distributions of responders and non-responders, as we used a convenience sample drawn from the northeastern United States, while Wood and colleagues [1] conducted their study in Australia, and Geffroy and coworkers [6] tested participants in France. However, there are also methodological differences to consider. As noted above, the participants in Wood and colleagues’ study [1] were employees or students at the Australian Wine Research Institute (AWRI). Presumably, these individuals are much more likely than naïve Pennsylvanian consumers to be familiar with peppery wines, given both the popularity of Shiraz in the Australian market and their occupational exposure to wine and its sensory evaluation. Other prior work suggests that exposure to an odor can lower the detection threshold for that odorant (reviewed in [23]). As such, AWRI employees may have lower thresholds for rotundone due to more familiarity with and exposure to the compound. Further, it is also possible that employment at a wine research institute may be self-selecting for individuals with greater olfactory expertise or interest relative to the typical consumer, resulting in a non-random sample (see discussion in [24]).

Based on our rejection thresholds of ~56 ng/L, the majority of our participants are not fond of added rotundone in red wine at any detectable concentration. Focus groups indicated that consumers who disliked rotundone described it as earthy, strong, bitter, or biting. We did, however, observe a drastic segmentation where a small percentage of our participants clearly and consistently preferred added rotundone, even at very high concentrations (up to 750 ng/L). Focus groups indicated these consumers appreciated the spice notes and felt that the rotundone imparted aromas balanced both the flavor and the sweetness. As previous work suggests that preference for peppery notes is associated with familiarity and wine expertise [6], it is not surprising that these individuals were the minority in our sample, as we did not specifically recruit wine experts or individuals who expressed a preference for or familiarity with peppery wines.

The 56 ng/L rejection threshold for participants who did not prefer added rotundone aligns with detection thresholds estimated via the ASTM method (37 and 73 ng/L for ortho- and retronasal assessment), and it falls within the confidence intervals for detection using the graphical method (the lower bounds were 39 ng/L for retronasal and 58 ng/L for orthonasal assessment). If we use a similar decision rule for determining responders and non-responders as we used for the rejection threshold (consistent responses for the top 3 rotundone concentrations), approximately 30% of participants in this study were anosmic to rotundone, which is similar to previously published work [1,6].

Focus groups provided a notable finding in that the term “peppery” was not the most common descriptor provided by our participants for rotundone in wine. This may again reflect that our sample was primarily comprised of less experienced wine consumers who may not be familiar with the terminology agreed upon by wine experts. (Indeed, the spiked wine was clearly peppery to members of the research team who are able to smell rotundone). Moreover, the term peppery was also not particularly appealing to our participants conceptually. Our participants seemed to describe peppery wines in two ways: the sensory terms *strong* and *spicy* paired with conceptual terms like *bold* and *flavorful* or the sensory concept of *balance* paired with the conceptual terms *traditional* and *sophisticated*. These might be alternative ways to market high rotundone wines to consumers, since this language may more accurately reflect consumer expectations and experiences.

The present work has some limitations that should be mentioned. Here we used only a single set of concentrations for the detection thresholds, and only a single threshold estimate was determined for each participant. Had we used an interleaved series of concentrations across participants, or had participants assessed more concentration levels across multiple days, we may have gotten a slightly better fit for the regression line in our graphical threshold method; similarly, additional test samples at intermediate concentrations may have resulted in smaller confidence intervals. On the other hand, the added precision from additional concentrations or replicates may not be worth the substantial labor, resources, and, most critically, the participant burden they would require. Furthermore, only a single base wine was used here: as threshold estimates can also vary across different types of wines, the values reported here might differ in other red wines. Regarding the rejection thresholds, the decision rule used to classify individuals as consistent responders might have conceivably excluded those who could in fact smell rotundone, but only enjoy it low concentrations just above threshold (i.e., those showing an inverted U function between concentration and preference concentrations); as a rule, rejection thresholds are not well suited to uncover this response pattern. Our sample was also unbalanced in terms of participant gender. We did not have any sex or gender specific hypotheses for rotundone, so we did not look for any such differences, as they would be underpowered. Still, given the potential for sex differences in olfactory sensitivity (e.g., [25]), future research should potentially revisit this question. Finally, due to resource limitations, we did not make any effort to confirm the rotundone concentrations via chemical analysis. That said, we have no reason to believe the concentrations delivered deviated from the amounts prepared by research staff. Indeed, given the similarity between our orthonasal threshold estimation and that of Wood et al. [1], we are relatively confident about the levels we determined. For the focus groups, it is again a limitation that only a single base wine and single wine with naturally occurring rotundone were used. These results may not generalize to other wines that are naturally high in rotundone and further research in other wine varieties is warranted.

## 5. Conclusions

Our data confirm previous work documenting a specific anosmia for rotundone and extends it to a population not previously tested. We found similar detection and rejection thresholds to extant studies and confirmed that rotundone in wine can be quite polarizing for those who can perceive it. For our population of consumers, the peppery note contributed by rotundone was neither preferable when tasting wine nor was it conceptually appealing. We also found a slightly greater proportion of non-responders compared to previous studies (~40% vs. 20–30%). This could be due to either biological differences across populations, or methodological differences between our study and the two prior studies examining the perception of rotundone in red wine [1,6].

Further, we found no differences between the detection thresholds for rotundone in red wine using orthonasal and retronasal delivery. This is particularly pertinent to the wine industry, as it suggests that the peppery odor obtained by sniffing a wine may be a good representation of how a consumer can expect that wine to taste in the glass. Additionally, there are clear implications for the experience of non-responders. While it is true that expectation strongly influences olfactory perception [26], unwitting rotundone-anosmic consumers who purchase a peppery wine may be disappointed to find that they are unable to detect a peppery flavor. Educating consumers about the possibility of having a specific anosmia might help to manage this potential disappointment, which may otherwise be unfairly blamed on the winemaker. Additional work is needed to see how disconfirmation of expectations may influence consumer satisfaction.

Separately, given the differences in proportions of anosmic participants across the three regions tested to date (Australia, France, and Northeastern USA), we suggest additional research is needed to deduce the genetic basis for this specific anosmia, which may allow more accurate assessment of the incidence of rotundone non-responders around the globe. Such information is both biologically interesting and commercially relevant given the rapid growth of wine consumption in emerging markets like India and China.

## Figures and Tables

**Table 1:** Age distribution of sample for Study 1.

Age	Sample (*n* = 109)	Proportion
22–24	12	11.3%
25–30	36	34.0%
31–35	25	23.6%
36–40	17	16.0%
41–45	14	13.2%
46–50	0	0%
51–55	2	1.9%
Not reported	3	–

**Table 2:** Age distribution of sample for Study 2.

Age	Sample (*n* = 86)	Proportion
22–24	17	19.76%
25–30	26	30.23%
31–35	19	22.1%
36–40	9	10.46%
41–45	14	16.28%
46–50	0	0%
51–55	0	0%
Not reported	1	–

## Supplementary Materials

The following are available online at https://www.mdpi.com/2072-6643/12/9/2522/s1. Table S1. Participant demographics for Study 1; Table S2. Participant demographics for Study 2.
