# Supplementary material for: Individual Differences in Thresholds and Consumer Preferences for Rotundone Added to Red Wine
Source: Nutrients. 2020 Aug 20;12(9):2522. doi: 10.3390/nu12092522 (PMC7551619; doi:10.3390/nu12092522)
Supplement: Supplementary file 1 [file nutrients-12-02522-s001.pdf]

Table S1. Participant demographics for Study 1.

Prompt	Sample (n = 104*)	Prevalence
How often do you consume wine?		
Never	3	2.88%
A few times a year	15	14.42%
Once a month	13	12.5%
2–3 times a month	35	33.65%
Once a week	19	18.27%
2–3 times a week	17	16.35%
4–6 times a week	1	0.96%
Every day	1	0.96%
How often do you consume beer?		
Never	10	9.61%
A few times a year	14	13.46%
Once a month	10	9.61%
2–3 times a month	30	28.85%
Once a week	21	20.19%
2–3 times a week	15	14.42%
4–6 times a week	4	3.85%
Every day	0	0%
How often do you consume liquor straight?		
Never	13	12.26%
A few times a year	41	39.42%
Once a month	18	17.31%
2–3 times a month	17	16.35%
Once a week	6	5.77%
2–3 times a week	7	6.73%
4–6 times a week	2	1.89%
Every day	0	0%
How often do you consume mixed drinks?		
Never	4	3.85%
A few times a year	38	36.54%
Once a month	21	20.19%
2–3 times a month	26	25%
Once a week	7	6.73%
2–3 times a week	7	6.73%
4–6 times a week	0	0%
Every day	1	0.96%

*5 participants did not provide this information.

Table S2. Participant demographics for Study 2.

Prompt	Sample (n = 85*)	Prevalence
How often do you consume wine?		
Never	1	1.18%
A few times a year	8	9.42%
Once a month	22	25.88%
2–3 times a month	20	23.53%
Once a week	13	15.29%
2–3 times a week	16	18.82%
4–6 times a week	4	4.71%
Every day	1	1.18%

How often do you consume beer?		
Never	9	10.59%
A few times a year	13	15.29%
Once a month	11	12.94%
2–3 times a month	10	11.76%
Once a week	20	23.53%
2–3 times a week	14	16.47%
4–6 times a week	8	9.42%
Every day	0	0%
How often do you consume liquor straight?		
Never	11	12.94%
A few times a year	25	29.41%
Once a month	16	18.82%
2–3 times a month	17	20%
Once a week	10	11.76%
2–3 times a week	4	4.71%
4–6 times a week	2	2.35%
Every day	0	0%
How often do you consume mixed drinks?		
Never	6	7.06%
A few times a year	22	25.88%
Once a month	16	18.82%
2–3 times a month	20	23.53%
Once a week	13	15.29%
2–3 times a week	6	7.06%
4–6 times a week	2	2.35%
Every day	0	0%

*1 participant did not provide this information.
